# Curcumin suppresses gastric cancer by inhibiting gastrin‐mediated acid secretion

**DOI:** 10.1002/2211-5463.12237

**Published:** 2017-06-21

**Authors:** Shufen Zhou, Dongjie Yao, Ling Guo, Ling Teng

**Affiliations:** ^1^ Department of Gerontology, Affiliated Second Hospital Mudanjiang Medical University China; ^2^ Department of Quality Control, Affiliated Second Hospital Mudanjiang Medical University China; ^3^ Department of Pathology, Affiliated Second Hospital Mudanjiang Medical University China

**Keywords:** acid secretion, curcumin, gastric cancer, gastrin

## Abstract

Hyperacidity in the stomach is known to promote the progression of gastric cancer. The plant‐derived chemotherapeutic curcumin is used to treat gastric cancer. The objective of this study was to investigate whether curcumin regulates gastrin‐mediated acid secretion in suppressing gastric cancer. Gastric cancer cells were treated with 25 μm curcumin, followed by Annexin V/propidium iodide double‐staining assay to evaluate cell apoptosis. Western blot analysis was used to analyze caspase‐3 expression in response to curcumin treatment. Gastrin levels in culture medium were also monitored. Mice bearing gastric cancers were treated with curcumin, followed by analysis of tumor caspase‐3 expression, gastric acid pH, and gastric secretion in serum. Curcumin prominently inhibited gastric cancer cell proliferation and promoted cell apoptosis. Caspase‐3 was upregulated by curcumin treatment. Curcumin also reduced gastrin secretion. Curcumin dramatically inhibited tumor growth, increased gastric pH, and reduced gastric secretion. In gastric cancer, curcumin suppresses gastrin‐mediated acid secretion, which inhibits gastric cancer progression.

**Abbreviations**
CCK‐8: cell‐counting‐Kit 8
HRP: horseradish peroxidase

Gastric cancer is one of the most common malignancies in the world 1. The 5‐year survival rate of gastric cancer is extremely low. Most gastric cancer patients are diagnosed at a high clinical stage, which demands potent treatment to impeded the disease progression and improve clinical outcomes. Curcumin is one of the chemotherapy drugs that have demonstrated strong efficacy in gastric cancer. Curcumin is a naturally occurring compound extracted from the rhizomes of plants in the ginger family 2. It has a broad‐spectrum therapeutic potential as a pharmaceutical agent, with antioxidative, anti‐inflammatory, and anticancer properties 3. However, despite efforts in elucidating the effects of curcumin in regulating oncogenic pathways 4, how curcumin exerts anticancer effects in gastric cancer still remains largely unknown.

It is increasingly realized that, as opposed the alkaline condition in other tissues, an acidic environment in stomach is conducive to gastric cancer survival and development 5. The development of gastric cancer is often accompanied by excessive secretion of acids 5, 6, 7. It is believed that low pH environment in stomach enables gastric cancer cells to evade immune attack. It also activates genes that promote cancer cell growth, and protects cancer cells against apoptosis 8, 9. Therefore, low gastric pH is thought of as a marker for high‐grade gastric cancers. Gastric acid secretion is stimulated by gastrin 10. This is supported by the increased serum gastrin levels in high‐level gastric cancer patients. This gave birth to a number of strategies to prevent cancer cell progression by reducing gastrin expression 11. Given the anticancer efficacy of curcumin, it is possible that curcumin also regulates gastrin expression in gastric cancer. However, no evidences exist concerning whether the efficacy of curcumin correlates to its regulation of gastric pH via gastrin secretion.

Therefore, in this study, we sought to correlate curcumin to the gastrin‐mediated gastric acid secretion and investigate how this inhibits gastric cancer. To this end, we evaluated how curcumin affects gastric expression in gastric cells *in vitro* and *in vivo*. We demonstrated that curcumin was able to serve as a gastrin antagonist. Our data also indicated that gastric pH in curcumin‐treated mice was higher. The data reported in this study could provide scientific ground for further clinical testing of curcumin in treating gastric cancer, strengthening the role of gastric pH in gastric cancer development.

## Materials and methods

### Cell culture and curcumin treatment

The human gastric cancer cell line, SGC7901, was acquired from American Type Culture Collection (ATCC, Rockville, MD, USA). Cells were cultured in the RPMI‐1640 medium supplemented with 10% FBS, 100 IU·mL^−1^ penicillin–streptomycin, 10 mm HEPES, and 2 mm glutamine. For all *in vitro* experiments, curcumin (Sigma‐Aldrich, Saint Louis, MO, USA) was dissolved in DMSO and added to the medium at the concentration of 25 μm.

### Cell proliferation assay

The proliferation of SGC7901 cells was evaluated with the cell‐counting kit 8 (CCK‐8) assay (Dojindo Molecular Technologies, Kumamoto, Japan) according to manufacturer's recommendations. Briefly, SGC7901 cells were first seeded into 96‐well plates (5 × 10^3^ cells per well). Treatment of curcumin lasted for 7 days with medium changed every 2 days. The control cells were treated with equivalent amount of DMSO. Cell viability was monitored every 2 days by incubating the cells with CCK‐8 solution for 4 h, followed by measuring absorbance at 450 nm.

### Annexin‐V/propidium iodide double‐staining assay

Cell apoptosis was evaluated by the Annexin‐V/propidium iodide double‐staining assay. Briefly, cells seeded in six‐well plates (1 × 10^5^ cells per well) were treated with curcumin. After 3 days of treatment, cells were harvested by trypsinization and washed with PBS. Staining of the cells was performed using the Annexin‐V/FITC apoptosis detection kit (Sigma, St. Louis, MO, USA) according to manufacturer's recommendations. Cell apoptosis was quantitated on a FACScan flow cytometer (Becton Dickinson, Franklin Lakes, NJ, USA).

### Western blot

Caspase‐3 expression was analyzed using western blot analysis to evaluate cell apoptosis. Lysates from cells were acquired after lysis in RIPA buffer (Thermo Fisher Scientific, Waltham, MA, USA). Quantitation of protein content was carried out using the bicinchoninic acid assay (Abcam, Cambridge, MA, USA). Protein (50 μg) was resolved using SDS/PAGE, followed by being electrotransffered onto PVDF membranes. Blocking was performed in 1% BSA solution. Primary antibodies for caspase‐3 and GAPDH were acquired from Abcam, which were used to incubate the membranes overnight at 4 °C. TBST (0.1% tween‐20) was used to wash the membranes. Appropriate horseradish peroxidase (HRP)‐conjugated secondary antibodies diluted in TBST were then added to the membrane. Bound antibodies were visualized using an enhanced chemiluminescence ECL regeant (Pierce, WI, USA) and imaging using the ChemiDoc imaging system (Abcam). Protein band intensities were quantitated using imagej (NIH, Bethesda, MD, USA), which were normalized to the level of GAPDH.

### Animal experiments

All animal experiments were performed in accordance with the National Institutes of Health Guide for the Care and Use of Laboratory Animals, and were approved by the Institutional Animal Care and Use Committee (IACUC) of Affiliated Second Hospital, Mudanjiang Medical College. The Balc/c nude mice were purchased from Nanjing Model Animal Center. SGC7901 cells (1 × 10^4^) were subcutaneously injected into the left foreleg to initiate gastric cancer xenografts 12. Curcumin dissolved in DMSO were orally administered into the mouse (100 mg·kg^−1^). The death, suffering, sick, moribund, and skinniness of the mice were documented to analyze survival. For measurement of gastric pH, mice were anaesthetized under isoflurane and stomachs were excised. The pH of gastric content was measured by immersing a pH probe in the gastric content. Mice undergone pH measurement were sacrificed immediately afterward experiments. After a 2‐month treatment, blood samples were collected by drawing from the heart and mice were sacrificed in a CO_2_ chamber. The tumor sizes were recorded. Blood samples were centrifuged at 1500 ***g*** for 15 min and stored in −20 °C before use. Gastrin levels in serum were analyzed with ELISA (Abcam) according to manufacturer's recommendations.

### Statistical analysis

All experiments were performed in triplicates. Data were represented as mean ± SD. Analysis of the data was performed using one‐ or two‐way ANOVA analysis followed by a post hoc test using the spss software (SPSS Inc., Chicago, IL, USA). The *P* value of less than 0.05 was considered statistically significant.

## Results

### Curcumin inhibits gastric cancer cell proliferation and induces cell apoptosis

**Figure 1:**
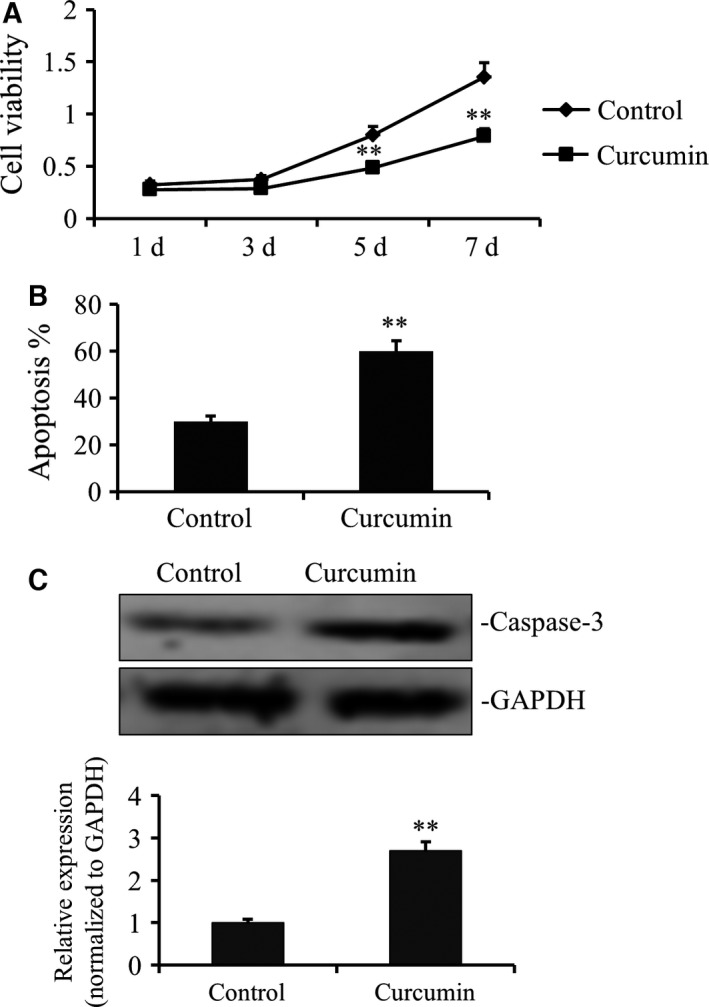
Curcumin suppressed the growth of gastric cancer cell line. (A) The SGC7901 cells were placed on 96‐well plates (1 × 10^4^ cells per well) and incubated with fresh medium as control group or containing 25 μm curcumin as treated group. Growth curves of individual curcumin‐treated and nontreated SGC7901 cells from the same original cell numbers were detected by CCK‐8 kit. OD value was measured at 450 nm and data demonstrated a significant growth decrease by curcumin (**P < 0.01). (B) Annexin‐V/propidium iodide double‐staining assay was performed to detect the apoptosis levels of curcumin‐treated and nontreated SGC7901 cells. The data demonstrated a significant apoptotic increase by curcumin (**P < 0.01). (C) Caspase‐3 gene expression of curcumin‐treated and nontreated SGC7901 cells was analyzed by WB. The data also demonstrated a significant apoptotic increase by curcumin (**P < 0.01).

Firstly, dose‐dependent effects of curcumin on the growth of gastric cancer cell line were studied. It was found that the dose of curcumin at 25 μm was the lowest dose to suppress the growth of gastric cancer cell line to the most extent (Fig. S1). Thus, the dose of 25 μm was chosen for the following studies. To further confirm the antitumor effects of curcumin, we incubated gastric cancer cells with 25 μm curcumin followed by evaluation of cell proliferation and apoptosis. As shown in Fig. 1A, significant inhibition on cell proliferation was observed. In line with this, curcumin‐treated gastric cancer cells also demonstrated higher apoptotic activity (Fig. 1B) based on Annexin V/propidium iodide double‐staining assay. Western blot analysis confirmed that capase‐3 level, which is positively correlated with apoptosis, was significantly upregulated compared to that of untreated cells (Fig. 1C).

### Acidification promotes gastric cancer cell proliferation and protects cells from apoptosis

**Figure 2:**
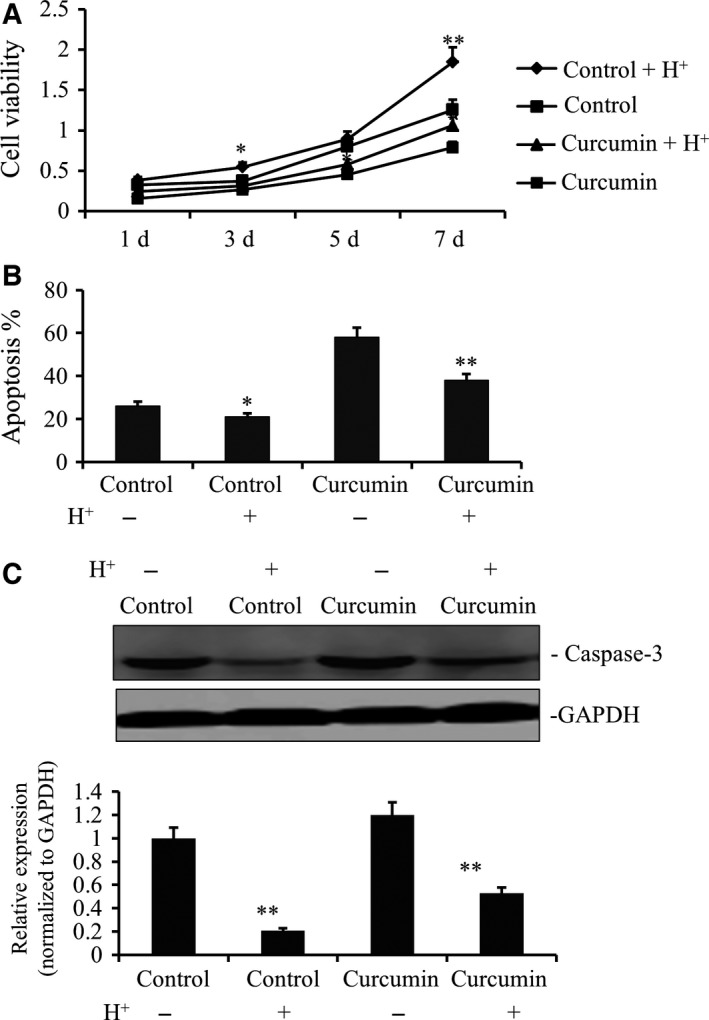
Acidity promoted the growth of gastric cancer cell line by the treatment with curcumin. (A) The SGC7901 cells were placed on 96‐well plates (1 × 10^4^ cells per well) and incubated with fresh medium as control group or containing 25 μm curcumin as treated group. Meanwhile, these two group of cells were, respectively, regulated to pH 6.4 by adding HCL solution. Growth curves of individual curcumin‐treated or not and acidulated SGC7901 cells or not from the same original cell numbers were detected by CCK‐8 kit. OD value was measured at 450 nm and data demonstrated a significant growth increase in acidic condition (**P < 0.01 and *P < 0.05). (B) Annexin‐V/propidium iodide double‐staining assay was performed to detect the apoptosis levels of curcumin‐treated or not and acidulated SGC7901 cells or not. (C) Caspase‐3 gene expression of curcumin‐treated or not and acidulated SGC7901 cells or not were analyzed by WB. The data also demonstrated a significant apoptotic decrease in acidic condition (**P < 0.01).

We then examined if low pH promotes gastric cancer survival and progression. We added hydrochloric acid to the cell culture medium to adjust the pH to 6.4. It was found that this acidified environment resulted in increased gastric cancer cell proliferation (Fig. 2A). However, incubation with curcumin, with acidification, led to lower gastric cancer cells proliferation (Fig. 2A). Meanwhile, while gastric cancer cells under acidification demonstrated lower apoptosis, as evidenced by lower apoptosis rate and caspase‐3 expression (Fig. 2B,C), treatment of curcumin counteracted this dampened apoptosis. Therefore, curcumin was able to potently antagonize the tumor‐promoting effects of acidification.

### Curcumin inhibits gastrin secretion

**Figure 3:**
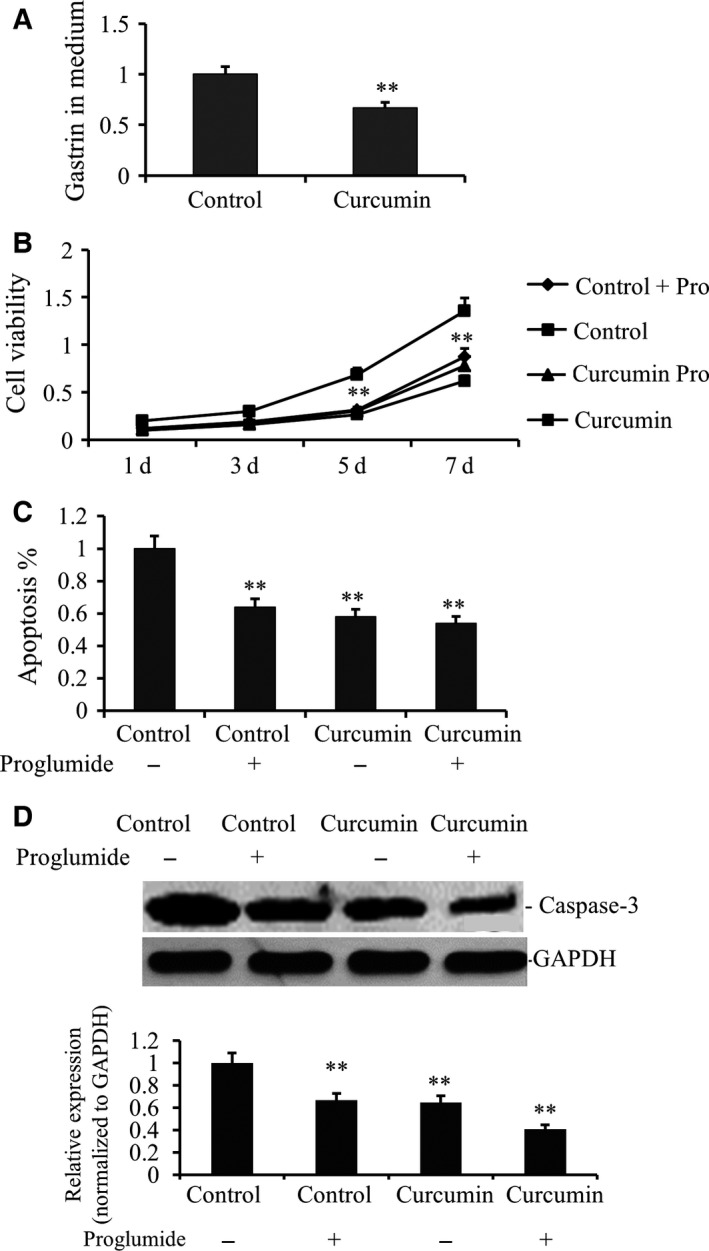
Curcumin suppressed the secretion of gastrin in gastric cancer cell line. (A) The SGC7901 cells were placed on six‐well plates (1 × 10^5^ cells per well) and incubated with fresh medium as control group or containing 25 μm curcumin as treated group. Gastrin level was detected by PCR in curcumin‐treated and nontreated SGC7901 cells after the culture for 48 h. Relative expression values represent mean and SD from three independent experiments (**P < 0.01). The data demonstrated a significant decrease in gastrin by curcumin. (B) Growth curves of individual curcumin‐treated or not and proglumide‐treated or not of SGC7901 cells from the same original cell numbers were detected. OD value was measured at 450 nm and data demonstrated a significant growth decrease by proglumide, but no difference for curcumin‐treated cells. (C) Annexin‐V/propidium iodide double‐staining assay was performed to detect the apoptosis levels of curcumin‐treated proglumide‐treated or not of SGC7901 cells. The data demonstrated a significant apoptotic increase by proglumide (**P < 0.01), but no difference for curcumin‐treated cells. (D) Caspase‐3 gene expression also was analyzed by WB. The data also demonstrated a significant apoptotic increase by proglumide (**P < 0.01), but no difference for curcumin‐treated cells.

Gastrin is a putative regulator of gastric acid secretion. As our data indicated an antagonizing role of curcumin against acidification, it is imperative to confirm if curcumin lowers gastrin secretion. To this end, gastrin secretion by gastric cancer cells under curcumin treatment was assessed. While gastrin was abundantly secreted by gastric cancer cells, gastrin level in the medium of curcumin‐treated cells was much lower than that in untreated cells (Fig. 3A). Following this, we added proglumide, a gastrin antagonist, into the culture medium. Expectedly, the proliferation of gastric cancer cells was also suppressed but the suppressive effect was weaker than that of curcumin. Interestingly, proglumide slightly compromised the antiproliferative ability of curcumin (Fig. 3B). This is possibly due to the fact that proglumide competed with curcumin in inhibiting gastrin. In agreement to this, Annexin V/propidium iodide double‐staining assay showed that apoptosis levels in proglumide‐treated gastric cancer cells were elevated, but curcumin‐treated gastric cancer cells did not show significant elevation of apoptosis after treatment with proglumide (Fig. 3C). Western blot analysis of gastric cancer cells in the apoptotic gene capase‐3 inhibition of gastrin confirmed that curcumin exerts its antitumor effects by promoting apoptosis (Fig. 3D).

### Curcumin inhibits of gastric cancer *in vivo*

**Figure 4:**
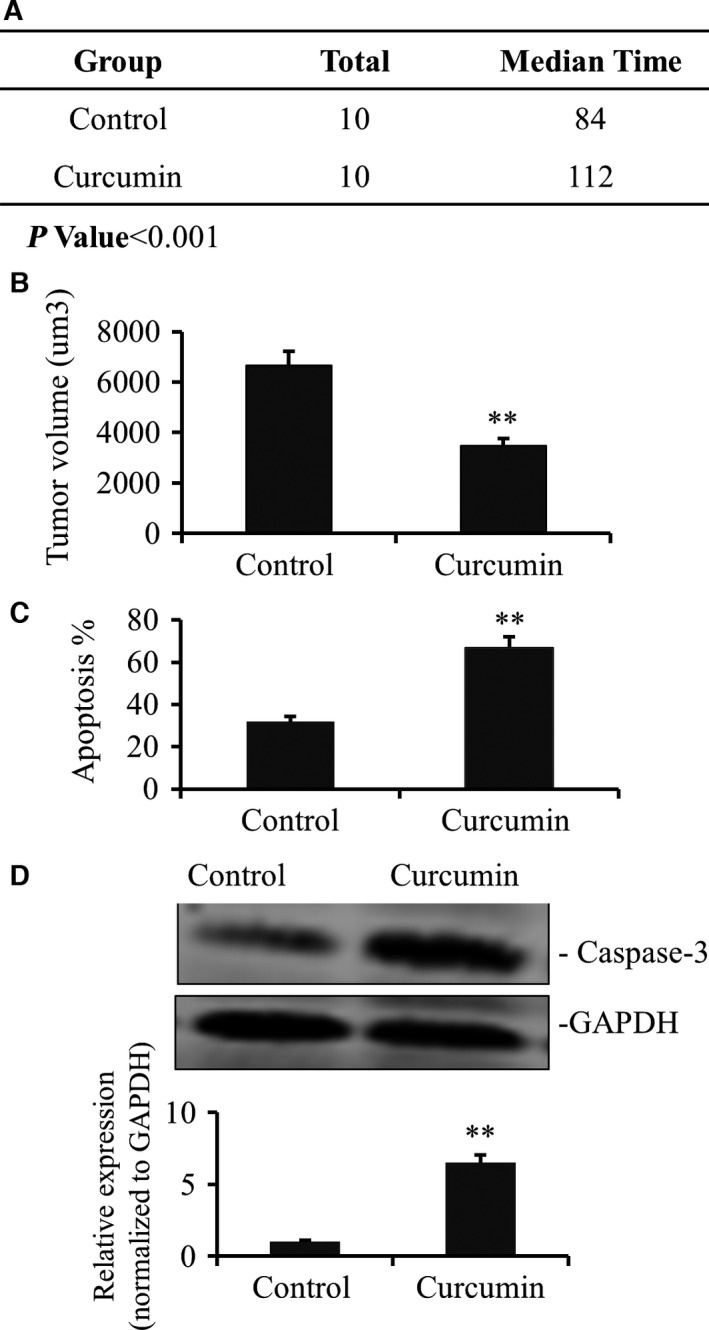
Curcumin suppressed the growth of gastric cancer *in vivo*. (A) The xenograft gastric cancer was established after injection 1 × 10^4^ different cells in the left foreleg. One group of mice was treated by curcumin (100 mg·kg^−1^). The survival times were calculated after each mouse was dead suffering sick, moribund, and skinny (*n* = 10, *P* < 0.001). (B) Tumor growth in xenografts inoculated from curcumin‐treated or nontreated models after 2 months while all the mice were still survival but sick. (C) Annexin‐V/propidium iodide double‐staining assay was performed to detect the apoptosis levels of curcumin‐treated or not of tumor tissue of models. The data demonstrated a significant apoptotic increase by curcumin (**P < 0.01). (D) Caspase‐3 gene expression also were analyzed by WB. The data also demonstrated a significant apoptotic increase by curcumin (**P < 0.01). Values represent mean and SD from three independent experiments.

Gastric cancer cell lines were subcutaneously injected into mice to initiate gastric cancer xenografts. Curcumin was given at the dose of 100 mg·kg^−1^ for 2 weeks and the tumor‐bearing mice without treatment were used as controls. Tumor growth was monitored and survival data of mice were acquired. We show that curcumin treatment led to a substantially higher survival (Fig. 4A). Consistently, tumors of curcumin‐treated mice were significantly smaller than those of untreated mice (Fig. 4B). Annexin V/propidium iodide double‐staining assay indicated that tumor apoptosis in curcumin‐treated mice was significantly higher than that in untreated mice (Fig. 4C). Capase‐3 was significantly higher in untreated tumor tissue than in untreated tumor tissue (Fig. 4D).

### Curcumin inhibits gastrin and gastric acid secretion

**Figure 5:**
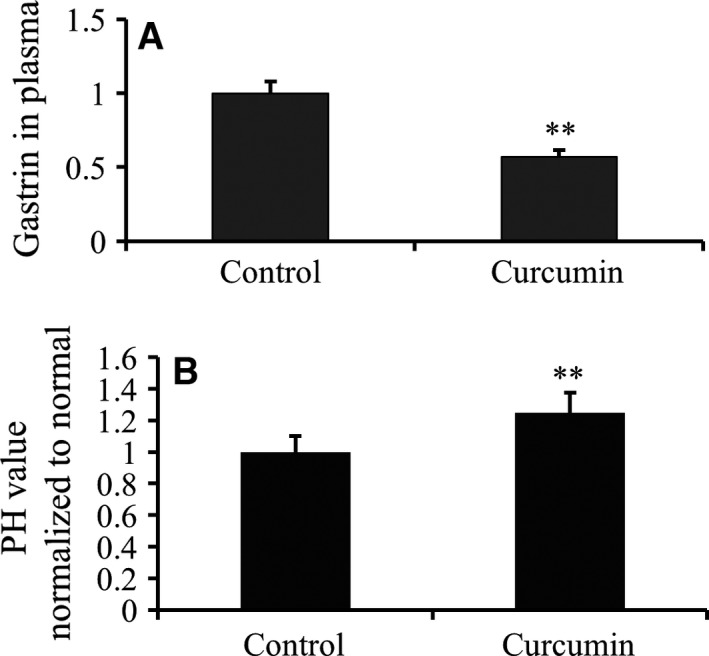
Secretion of gastric acid and gastrin decreased by treated with curcumin *in vivo*. (A) The xenograft gastric cancer was established after injection 1 × 10^4^ different cells in the left foreleg. One group of mice was treated with curcumin (100 mg·kg^−1^). The plasma gastrin was detected of curcumin‐treated or not mice after 2 months while all the mice were still survival but sick. Values are mean and SD from at least three independent experiments in duplicate (*n* = 6) (**P < 0.01). (B) Meanwhile, the pH values in gastric juice of curcumin‐treated, or not, mice were tested. The data showed pH values of gastric juice was increased by curcumin (**P < 0.01).

During the animal study, serum gastrin was monitored. We show that with curcumin treatment, gastrin secretion was significantly reduced (Fig. 5A). Concomitantly, the pH value of gastric fluid of the curcumin‐treated mice was significantly higher than that of the untreated mice (Fig. 5B). This confirms that curcumin lowers gastrin secretion to elevate gastric fluid pH, which inhibits gastric cancer progression.

## Discussion

Curcumin is a polyphenol with excellent safety, tolerability, and nontoxicity that have been demonstrated in clinical trials. Thus far, curcumin has shown therapeutic potential in a wide range of human disorders 13. Here, we corroborated that curcumin potently inhibits gastric cancer growth both *in vitro* and *in vivo*, accompanied by significant gastrin downregulation and hypoacidity in stomach. Previously, the potential of curcumin in regulating gastric cancer was thought attributable to downregulation of oncogenic pathways, such as PAK1 and cyclin D1 pathways 2. Curcumin was also shown to reverse chemoresistance of gastric cancer by downregulating the NF‐ĸB pathway 14. These findings are consistent with the role of curcumin as an immunosuppressant by regulating cytokine production and immunological gene pathways to inhibit cancer progression.

Thus far, whether curcumin affects acid production in gastric cancer is unclear. It is increasingly realized that the acid environment in stomach promotes gastric cancer. Previously, the link between gastric acid and bacteria‐induced gastric diseases has been established. Hyperacidity causes injury to stomach, and in severe conditions, ulcer may develop. Infections in the stomach may result in chronic inflammation that increases risk of carcinogenesis. In addition, hyperacidity was shown to promote *Helicobacter pylori* infection, which is a significant cancer inducer 15. Increased gastric juice acidity is also associated with higher risk of hiatabl hernia‐related gastric diseases 16. In cancer, low pH environment favors the formation of hypoxia, which is well‐known for conferring cancer resistance to chemotherapies 17. Indeed, our data showed an increased proliferation of gastric cancer cells under low pH. The inducer of acid secretion, gastrin, is proven a valuable biomarker in the screening of gastric cancer 18, 19. Gastric cancer cells overexpress gastrin as a way to remodel microenvironment to facilitate the outgrowth and metastases. Our data indicate that gastrin secretion is markedly inhibited by curcumin treatment. This is consistent with a recent study showing that curcumin protects against gastric mucosa by inducing hypoacidity in the stomach 20. Our *in vivo* data also indicate that gastric pH is significantly increased during curcumin treatment. To our knowledge, this is the first time that the anticancer effects of curcumin have been associated with its effects in inducing hypoacidity in stomach. It is also worth noting that curcumin is better tolerated than other acid suppressive drugs in that curcumin treatment is not associated with adverse effects, such as pneumonia 21, commonly seen for other acid suppressive drugs.

We showed that curcumin reduces gastrin secretion by gastric cancer cells. Similar to proglumide, curcumin acts as an antagonist to gastrin and curcumin demonstrated superior anticancer efficacy in gastric cancer compared to proglumide. In gastric cancer, gastrin is responsible for not only inducing acid secretion but also stimulating a number of oncogenic pathways, such as cyclin D1 and β‐catenin pathways 22, to induce cancer progression. Therefore, it can be speculated that by suppressing gastrin secretion, curcumin disrupts a cascade of cancer‐related events to impede the development of cancer. In line with this, we demonstrated that higher apoptotic activity was seen in curcumin‐treated gastric cancer cells. This echoes with the clinical data showing that patients treated with curcumin demonstrated lower gastrin secretion, as well as decreased tumor burden. Our study is paralleled by efforts in developing gastric antagonists or gastrin receptor antagonists to retard tumor progression 11. Besides, gastrin also plays an important role in other cancers, such as pancreatic cancer 23 and other malignancies, such as natriuresis and diuresis 24. By suppressing gastrin expression, curcumin should also exert ameliorating effects in these malignancies as well.

Currently, curcumin is not yet tested in clinical treatment of gastric cancer. Our study demonstrated that cancer cell proliferation was inhibited and apoptosis activity was increased by curcumin treatment. Our data extend our knowledge on the mechanism of curcumin in retarding gastric cancer progression, and could possibly give rise to new applications of curcumin based on its effects in regulating gastrin and gastric pH. Its nontoxicity and great tolerability also potentiate it as a chemopreventative drug and its regulation on immunological response, oxidative stress, etc., could also benefit patients with nonlethal diseases, such as hiatabl hernia and *H. pylori* infections.

## Author contributions

SZ, DY, and LG acquired the data, analyzed, and interpreted the data. LT conceived, designed the project, and wrote the paper.

## Supporting information


**Fig. S1.** Curcumin dose‐dependent suppressed the growth of gastric cancer cell line.
