# Supplementary material for: Curcumin suppresses gastric cancer by inhibiting gastrin‐mediated acid secretion
Source: FEBS Open Bio. 2017 Jun 21;7(8):1078–84. doi: 10.1002/2211-5463.12237 (PMC5537064; doi:10.1002/2211-5463.12237)
Supplement: Supplementary file 1 — Fig. S1. Curcumin dose‐dependent suppressed the growth of gastric cancer cell line. [file FEB4-7-1078-s001.docx]

**Supplementary Figures**


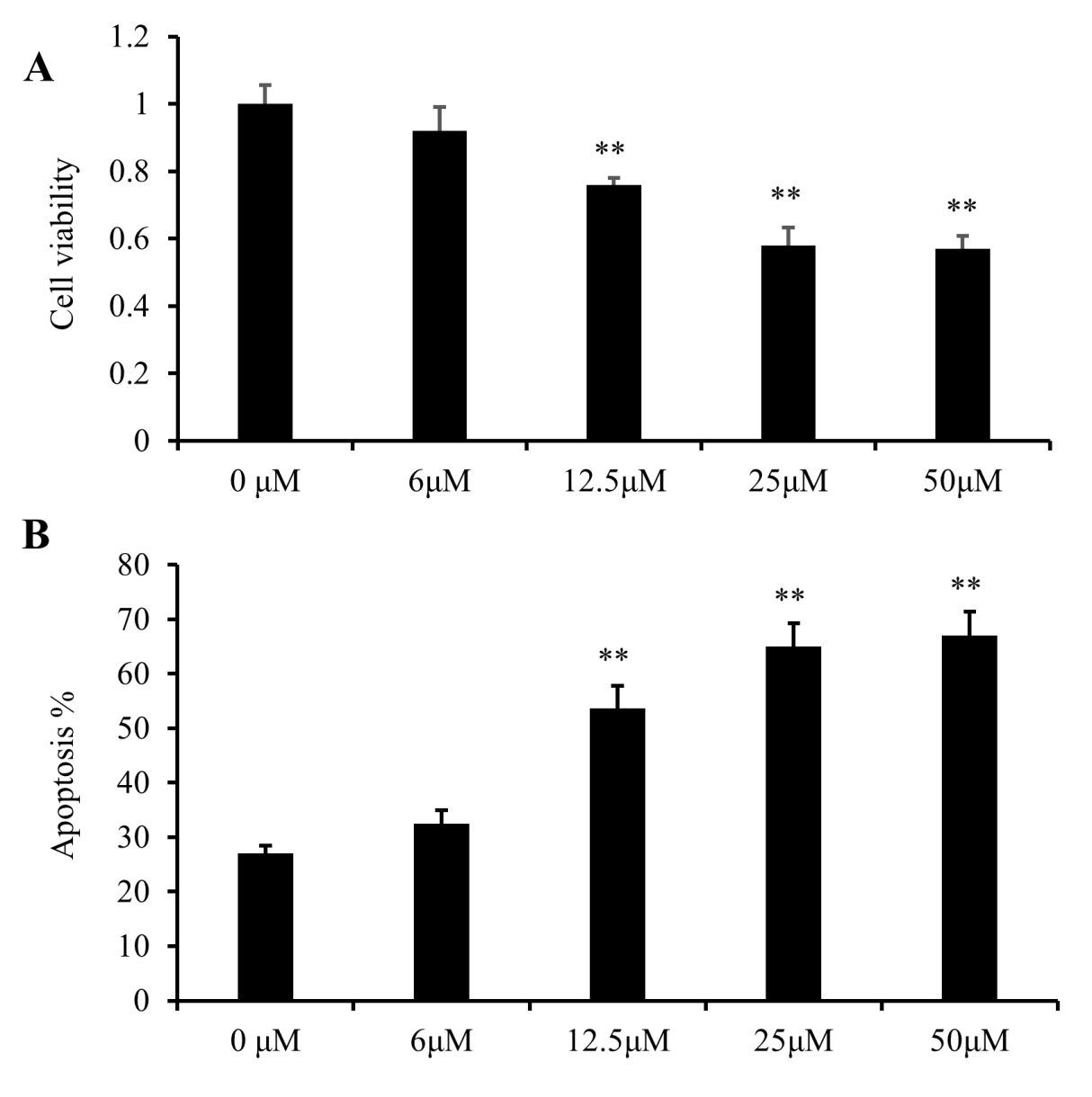


**Supplementary Figure S1. Curcumin dose-dependent suppressed the growth of gastric cancer cell line.** The SGC7901 cells were placed on 96-well plates (1×10^4^ cells/well) and incubated with fresh medium as control group or containing 6, 12.5, 25, 50 μM curcumin as treated group. Growth curves of individual curcumin-treated and non-treated SGC7901 cells from the same original cell numbers (1×10^4^ cells in 96 well-plates) were detected for treatment 72h at least three independent experiments in triplicate by CCK-8 kit (*p<0.01*). **B**. Annexin‑V/propidium iodide double-staining assay was performed to detect the apoptosis levels of curcumin-treated (6, 12.5, 25, 50 μM) and non-treated SGC7901 cells for treatment 72 h. Relative expression values represent mean and SD from three independent experiments (*p<0.01*).
